# A simulation-based analysis of optical read-out for electrochemical reactions using composite vortex beams

**DOI:** 10.1038/s41598-024-72701-8

**Published:** 2024-09-27

**Authors:** Nirjhar Kumar, Ankit Arora, Ananth Krishnan

**Affiliations:** Centre for NEMS & Nanophotonics CNNP and Department of Electrical Engineering, Indian Institute of Technology Madras, Chennai, 600036 India

**Keywords:** Imaging and sensing, Integrated optics, Imaging studies

## Abstract

We propose an optical read-out method for extracting faradaic current in electrochemical (EC) reactions and analyze its performance using opto-EC simulations. Our approach utilizes structured electrodes to generate composite optical vortex (COV) beams upon optical illumination. Through opto-EC simulations, we demonstrate that the EC reaction of 10 mM potassium ferricyanide induces a refractive index (RI) change, $\Delta$RI, of approximately $10^{-4}$ RI units, leading to the rotation of the COV beam’s intensity profile with a peak rotation of $40^{\circ }$. This rotation’s magnitude is proportional to $\Delta$RI, while the rate correlates with the faradaic current ($I_f$) density responsible for $\Delta$RI. As the opto-EC information is from bulk $\Delta$RI, it remains unaffected by interfering non-faradaic components at the interface and is advantageous for studying intermediate species and bulk homogeneous reactions. Furthermore, as rotation depends on $I_f$ density rather than $I_f$ itself, this method proves beneficial in low $I_f$ scenarios, such as when employing micro-electrodes to decrease solution resistance or obtain localized EC data. Even in low $I_f$ density scenarios, like monitoring slow EC reactions, our method enables signal amplification by accumulating rotation over time. This interdisciplinary approach holds promise for advancing EC research and addressing critical challenges across various fields, including energy storage, corrosion protection, environmental remediation, and biomedical sciences.

## Introduction

**Fig. 1:**
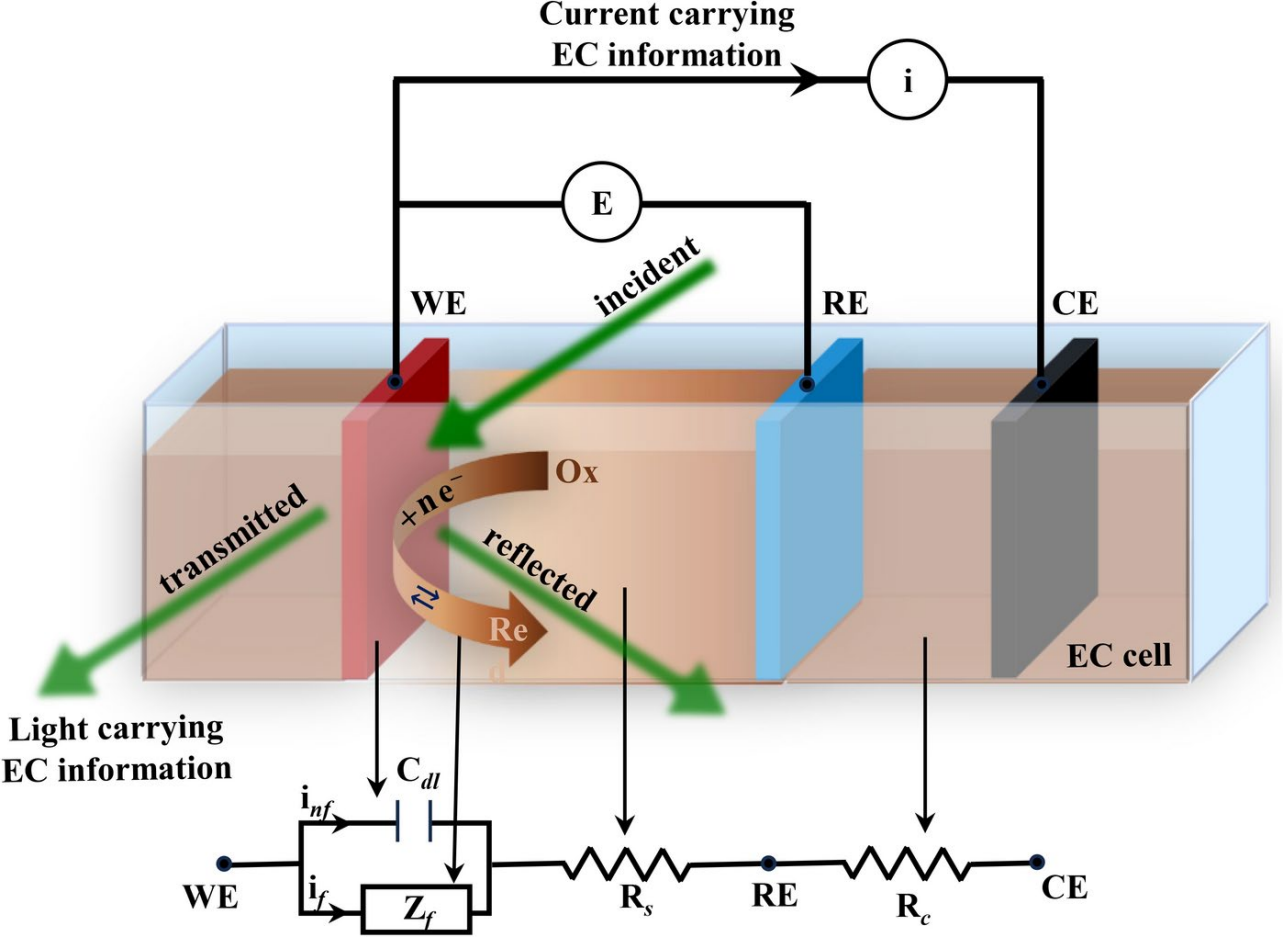
The diagram illustrates the schematic of an O-EC setup, featuring a conventional 3-electrode EC cell with a working electrode (WE), reference electrode (RE), and counter electrode (CE). Below the EC cell, the Randles equivalent circuit model is shown, including series resistance ($\hbox {R}_s$), double-layer capacitance ($\hbox {C}_{{dl}}$), and faradaic impedance ($\hbox {Z}_f$) between WE and RE, along with series resistance ($\hbox {R}_c$) between RE and CE. The voltmeter connections signify electrical excitation (**E**) for an EC reaction of the redox couple Ox and Red, while the ammeter symbolizes electrical probing of the resultant current (**i**). The green arrows represent optical probing.

Electrochemical (EC) reaction analysis is essential for understanding fundamental chemical processes and designing efficient EC systems^1,2^. Examining electron behavior at electrode–electrolyte interface can reveal insights into reaction mechanisms, characterize electroactive species, and optimize reaction conditions^1–4^. This knowledge is pivotal in various fields, including energy storage, corrosion protection, environmental remediation, and biomedical applications^5–18^. An EC setup consists of an EC cell and a potentiostat. A typical three-electrode EC cell, illustrated in Fig. 1, comprises a working electrode (WE), a reference electrode (RE), and a counter electrode (CE) immersed in an electrochemically active solution known as the electrolyte. These electrodes are connected to a potentiostat, which applies a driving potential **E** across WE and RE while measuring the resulting current **i** between WE and CE, or vice versa. One component of interest in **i** is the faradaic current ${\textbf {i}}_f$, which carries information about the EC reactions occurring at the WE. The other component, a non-faradaic current ${\textbf {i}}_{{nf}}$, often not of interest, includes the charging currents associated with the double-layer capacitor at the electrode-electrolyte interface, depicted as $\hbox {C}_{{dl}}$ in the Randles equivalent circuit model in Fig. 1. In a similar manner, **E** also comprises two components. The Ohmic drop component ${\textbf {E}}_R$ represents the potential lost during transmission from RE to WE due to the solution resistance $\hbox {R}_s$. As a result, the other component, the effective potential ${\textbf {E}}_{{eff}}$ available for the EC reaction is diminished from **E**.

Among the array of EC techniques, cyclic voltammetry (CV) is one of the most extensively employed methods owing to its simplicity and versatility in exploring a diverse spectrum of EC phenomena^19,20^. CV involves sweeping **E** linearly with time and analyzing **i**, focusing on the shape, position, and magnitude of peaks in **i** to gain insights into reaction mechanisms, kinetics, and thermodynamics^19–21^. However, this analysis is complicated by the presence of ${\textbf {i}}_{{nf}}$ and ${\textbf {E}}_R$^22^. When these non-faradaic components are dominant, the CV plot may become highly distorted, potentially masking an accurate interpretation of EC behavior^23,24^.

Some of the conventional approaches to mitigate ${\textbf {i}}_{{nf}}$ involve background current subtraction^1,24^, various forms of pulse voltammetry^1,25^, and AC voltammetry^1,26^. Numerous signal post-processing techniques, including those based on Fourier domain analysis, convolution-based methods, and independent component analysis, have been suggested for ${\textbf {i}}_{{f}}$ recovery^27–30^. Several variations of **E** waveform have also been proposed to optimize the extraction of EC reaction parameters^31,32^. Whereas, the conventional approaches to mitigate ${\textbf {E}}_{R}$ loss include improving the electrolyte’s conductivity through the addition of supporting electrolytes and minimizing the distance between WE and RE using a Luggin probe or micro-electrodes^1,33^. Another approach involves external compensation using a potentiostat^34^. Furthermore, for a comprehensive understanding of EC systems, particularly in studying homogeneous bulk reactions or intermediate species produced at the WE, it is crucial to distinguish between processes occurring at the electrode–electrolyte interface and within the bulk electrolyte. This distinction is often achieved through functional tagging^35^ or EC methods such as rotating disk electrode (RDE) or rotating ring-disk electrode (RRDE) setups, combined with numerical simulations or theoretical models^36^.

All the aforementioned methods address the issue with electrical signals **E** and **i** within the realm of the electrical domain. However, upon transitioning the signal to the optical domain, the original problem may cease to exist in the new domain, thereby eliminating the necessity for additional intervention. Moreover, exploring the optical domain can provide additional insights and complementary data to traditional EC measurements, contributing to a deeper and more comprehensive understanding of EC processes. Additionally, the optical domain grants access to a wide array of optical tools and techniques. This transition of signal domain can be achieved using an Opto-electrochemical (O-EC) setup, where optical beams are incident on or near the WE and the captured reflected or transmitted beams contain EC information, as depicted in Fig. 1 .

**Fig. 2:**
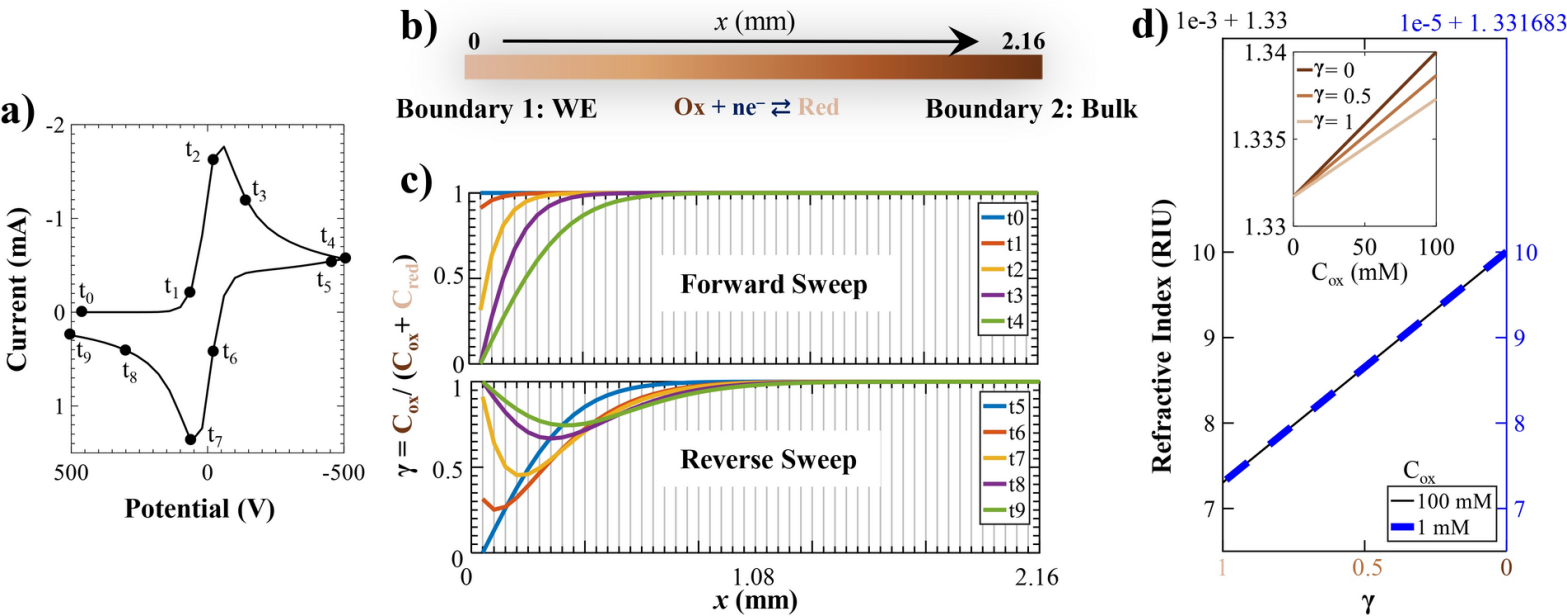
The figure illustrates how an EC reaction triggers a change in concentration, which in turn influences an optical property, namely the RI, thereby suggesting the potential for optical tracking of EC reactions. (**a**) Shows a typical CV plot obtained from simulation. (**b**) Shows the 1D CV simulation space where point x = 0 is the WE and the other end, x = 2.16 mm, is taken as the bulk. The color gradient between these two ends represents a possible concentration gradient/profile when a potential is applied on the WE. (**c**) Shows the normalized concentration $\gamma$ profile of the reactant at 10 different time instants corresponding to ten different points marked in the plot in (**a**). (**d**) Shows the variation of RI of the electrolyte with $\gamma$ for two different initial reactant concentrations. The inset plot shows the effect of initial reactant concentration on the RI for three different values of $\gamma$.

Different O-EC methods involve the use of surface plasmon resonance (SPR) spectroscopy, spectroscopic ellipsometry, Raman spectroscopy, or optical interferometry methods in an EC setup^37,38^. SPR spectroscopy involves detecting changes in the refractive index (RI) of a thin electrolyte film at the electrode surface induced by the reaction. While SPR provides sensitive detection of surface reactions, it may not fully capture bulk EC processes^39^. Spectroscopic ellipsometry measures changes in the polarization state of light upon interaction with the electrolyte, whereas Raman spectroscopy offers insights into molecular vibrations and can identify chemical species present in the electrolyte or at the electrode surface^40–42^. However, their application to EC analysis can be challenging due to interference from background signals and the need for careful calibration and understanding of the system^43^.

Optical interferometry techniques offer numerous advantages over other optical methods, particularly in their ability to provide high sensitivity and resolution for studying various phenomena. One key advantage is their capability to measure changes in optical path length with exceptional precision, enabling the detection of subtle variations in RI or sample thickness^44^. Nonetheless, a common drawback of all O-EC methods is the requirement for a bulky setup with precise optical alignment, which can pose several challenges^45^.

Therefore, we propose utilizing a structured metal grating to serve both as electrodes and as an on-chip compact interferometer simultaneously. These structured electrodes, under simultaneous electrical excitation and optical illumination, generate structured optical beams that carry information about the EC reaction within their intensity profile. We simulate the proposed method in three stages: (i) conducting CV simulations of a one-electron reversible electrochemical reaction (1e-RER), (ii) employing rigorous coupled-wave analysis (RCWA) for Fourier domain electromagnetic simulations of the structured electrode, and (iii) using Huygens’ principle for simulating the intensity profile of the structured beam. Through these simulations, we demonstrate that the resulting structured beam is a composite optical vortex (COV) beam that rotates with the progression of the EC reaction. We observed a peak rotation of $40^{\circ }$ for 1e-RER of a 10 mM aqueous solution of potassium ferricyanide and that the rate of COV beam rotation is directly proportional to $i_f$ per unit electrode area. The benefit of the proposed O-EC method in simplifying the analysis of EC systems dominated by non-faradaic components was emphasized through a direct comparison between the conventional CV plot ($\hbox {CV}_E$) and the corresponding optically obtained CV plot ($\hbox {CV}_O$). While the faradaic peak was indistinct in the $\hbox {CV}_E$ plot, it was clearly visible in the $\hbox {CV}_O$ plot. We conclude the discussion by highlighting the advantages and future prospects of the O-EC method, as well as addressing some of its limitations.

## Results

### CV simulation

Figure 2a shows a CV plot obtained from simulation for the 1D schematic shown in Fig. 2b. The simulation schematic for the 1e-RER used in this work has been discussed in detail in previous report^26^, and the simulation parameters are listed in Supplementary S1 as Table ST1. In an EC reaction, reactants are consumed at the WE and converted to products. These products diffuse away from the WE, while reactants diffuse toward the WE. This alters the product to reactant concentration ratio in the vicinity of the WE. The normalized reactant concentration $\gamma (x,t)$, i.e., the reactant concentration $c_{ox}(x,t)$ normalized with respect to the initial concentration $c_{ox}^*$ ( = $c_{ox}(x,t)$ + $c_{red}(x,t)$ ), is shown in Fig. 2c for different time instants marked in Fig. 2a. If the reactant and the product have different RIs, this change in reactant ratio causes a change in the RI of the electrolyte near the WE viz. in the depletion layer and the diffusion layer. The refractive index (RI) can be expressed in terms of $\gamma$ and for a given $C_{ox}^*$ using the Lorentz–Lorenz equation^46^, as given by Eq. (1) derived in Supplementary S1 as Eq. (S.4). For the simulation of a potassium ferri-ferro redox couple, where *ox* is $[\hbox {Fe(CN)}_6]^{3-}$ and *red* is $[\hbox {Fe(CN)}_6]^{4-}$, the assumed refractive index (RI) of water $RI_w$ and the values of polarizability $\alpha _{ox}$ and $\alpha _{red}$ derived from the work of Kragt et al.^47^ are provided in Supplementary S1 as Table ST1. Figure 2d demonstrates a linear dependence of RI on $\gamma$ which is similar for the two different $C_{ox}^*$ of potassium ferricyanide, with the only difference being the scale for the two concentrations. Furthermore, as expected from Eq. (1), a similar linear dependence of RI on $C_{ox}^*$ was observed and can be seen from the inset-plot for three different values of $\gamma$.

1 $$\begin{aligned} RI_{sup} = RI_w + 36.572\times 10^{36} C_{ox}^*[ (1-\gamma )( \alpha _{red}-\alpha _{ox}) + \alpha _{ox} ] \end{aligned}$$

### RCWA simulation

**Fig. 3:**
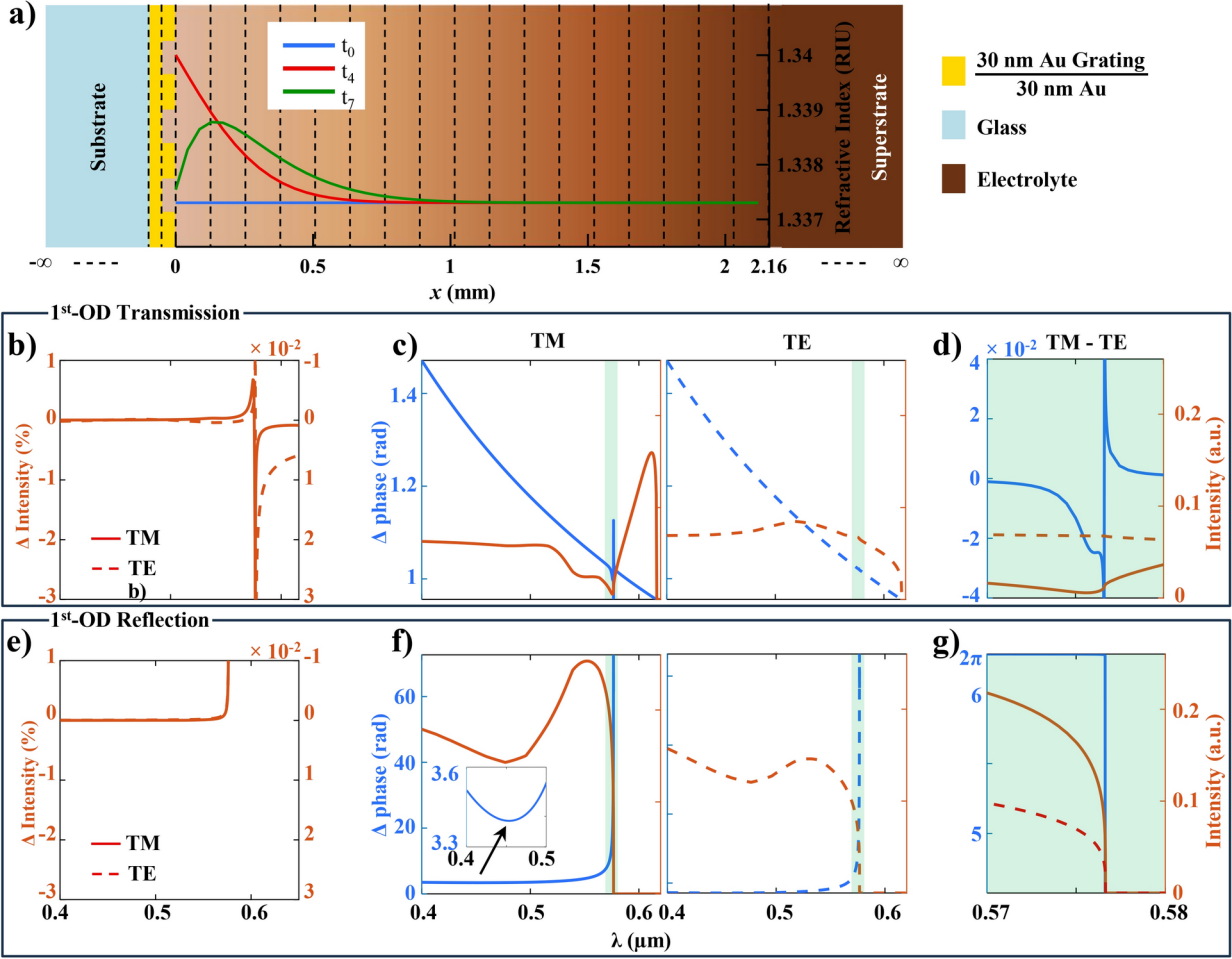
The figure illustrates how the change in the RI profile, induced by the EC reaction, can be inferred from the alteration in the optical phase, particularly the phase of the $1^{st}$-DO of a grating. (**a**) Shows the 2D RCWA simulation space derived from the 1D CV simulation space in Fig. 2b by dimensional extrusion along the y-axis. The concentration-dependent RI profile along the x-axis is discretized and assigned to different layers stacked along the x-axis. The WE is expanded into two layers, Au and Au-grating layer. The RCWA simulation space comprises these layers sandwiched between semi-infinite Glass (substrate) and bulk electrolyte (superstrate) layers. The plot shows the RI profile of the electrolyte at three different time instants corresponding to the three points marked in the CV plot in Fig. 2a. (**b**) Shows the % change in transmittivity for $\Delta RI_{sup}$ = $10^{-4}$ RI Units is $<<$ 1$\%$ for both TM and TE polarization. (**c**) Shows the change in phase of the transmitted $1^{st}$-DO for $\Delta RI_{sup}$ = $10^{-4}$. For all wavelengths except a band of $\lambda$ around the grating resonance at 576.8 nm (highlighted as a light green band), to till cut-off at 616 the change in phase for both TM and TE polarization is an identical monotonically decreasing function of $\lambda$. (**d**) Shows the difference in phase-change between TE and TM polarization in the grating resonance band, which is of the order of $10^{-2}$ and saturates beyond this band. (**e**–**g**) Shows the corresponding plots for the reflected $1^{st}$-DO.

Due to the linear relationship between the RI and $\gamma$ as shown in Fig. 2d , the RI profile shown in Fig. 3a is similar in nature to the $\gamma$ profile shown in Fig. 2c at the corresponding time instant. The RI profile variation can be detected through changes in the phase or any other optical parameters of an optical beam passed through the electrolyte. Our approach involves utilizing the $1^{st}$ diffraction order (DO) of a grating to track the phase change corresponding to variations in RI, with further rationale detailed in subsequent sections. The transmittance(*T*)/reflectance($\Gamma$) of the grating is simulated using 2D RCWA, with simulation parameters detailed in Supplementary S1 as Table ST1. The schematic for RCWA simulation is illustrated alongside relevant RI profiles in Fig. 3a. An inert metal, gold, was selected as the grating material to concurrently serve as electrodes for an EC reaction. In-line with our previous experimental report^48^, an ultra-thin metal grating on metal (ut-MGM) structure was simulated with parameters specified in Supplementary S1 as Table ST1. This setup allows for phase analysis in both reflection and transmission, as well as the exploration of plasmonic or grating resonance effects. The electrolyte is divided into multiple RCWA layers parallel to the electrode surface, with the RI of each layer determined as the mean RI on both layer planes. The RI of the electrolyte in the grating layers, i.e., within the grating grooves was assumed to be the RI at x = 0, i.e., at the WE. The RI of the superstrate was taken as the RI at x = 2.16 mm, i.e., the RI of the bulk. We examined the change in phase ($\Delta$phase) for the change in refractive index ($\Delta$RI) of the electrolyte for different wavelengths in the visible spectrum. The visible spectrum was chosen to enable the visual readout of an EC response.

2 $$\begin{aligned} \lambda _{gr} = P_G \Biggl [ 1 + \Bigl \{RI_{sup}-1\Bigl \} \times \Bigl \{0.5 - 0.1216\log (RI_{sup})\Bigl \} \Biggl ] \end{aligned}$$

Figure 3b–g shows the the effect of a uniform RI change $\Delta RI_{sup}$ = 1$\times 10^{-4}$ RI Units (RIU) within a uniform RI profile $RI_{sup}$ = 1.33 RIU across all layers on the intensity and phase of the $1^{st}$-DO in both reflection and transmission. The dashed line plot represents transverse electric (TE) polarization, while the solid line plot represents transverse magnetic (TM) polarization. From the intensity variation plot in Fig. 3b,e, it can be observed that $\Delta$intensity remains negligible ($<<$ 1%) for almost all wavelengths and across all cases. However from the phase variation plots, significant variations in $\Delta$phase are evident, prompting detailed examination. Each phase plot includes intensity in red, alongside $\Delta$phase in blue. Based on the grating resonant wavelength $\lambda _{gr} = 576.8$ nm calculated in the empirical equation Eq. (2) for the grating period $P_G$ = 500 nm, the phase plot analysis can be divided into three bands. A grating resonance band, highlighted in light green, comprises a thin wavelength band around $\lambda _{gr}$, sandwiched between a no-resonance band on the left and a plasmonic resonance band (with asymmetric resonant peak intensity at $\lambda _{pr}$ = 612 nm, cutoff at 616 nm) on the right.

3a $$\begin{aligned} \Delta \text {phase}_{inc}&= 2\pi x_{bulk} \frac{ \Delta RI_{sup}}{\lambda } \frac{P_G}{2 \lambda _{gr}} \end{aligned}$$

3b $$\begin{aligned} \Delta \text {phase}_{ref}&= 2\pi \frac{x_{bulk}}{\cos (\sin ^{-1}{(\lambda /\lambda _{gr}}))} \frac{ \Delta RI_{sup}}{\lambda } \frac{P_G}{2 \lambda _{gr}} \end{aligned}$$

3c $$\begin{aligned} \Delta \text {phase}_{op}&= {\left\{ \begin{array}{ll} \Delta \text {phase}_{inc}& \text { for 1-DO}_T \\ \Delta \text {phase}_{inc}+\Delta \text {phase}_{ref} & \text { for 1-DO}_\Gamma \end{array}\right. } \end{aligned}$$

3d $$\begin{aligned} \Delta \text {phase}&= {\left\{ \begin{array}{ll} \Delta \text {phase}_{op}+\Delta \text {phase}_{gr}+\Delta \text {phase}_{pr}& \text {for 1-DO}_T \\ \Delta \text {phase}_{op}+\Delta \text {phase}_{gr} & \text {for 1-DO}_\Gamma \end{array}\right. } \end{aligned}$$

In the no-resonance band, $$\Delta$$phase primarily arises from the phase difference ($$\Delta$$$$\hbox {phase}_{{inc}}$$) due to the change in the optical path-length of the incident beam traversing through the electrolyte. $$\Delta$$$$\hbox {phase}_{{inc}}$$ exhibits a monotonically decreasing trend with lambda, as shown in Fig. 3c and described in Eq. (3a). On the other hand, in reflection, additional $$\Delta$$$$\hbox {phase}_{{ref}}$$ given by Eq. (3b) accumulates due to changes in the optical path of the reflected beam traversing back through the electrolyte. As lambda increases, the path of the reflected $$1^{st}$$-DO becomes increasingly off-axis, resulting in a longer path inside the electrolyte, eventually approaching $$\infty$$ at the $$\lambda _{gr}$$ where the reflected path is at a grazing angle to the grating surface. The overall $$\Delta$$phase due to optical path difference ($$\Delta$$$$\hbox {phase}_{{op}}$$) for reflection encompasses both $$\Delta$$$$\hbox {phase}_{{inc}}$$ and $$\Delta$$$$\hbox {phase}_{{ref}}$$ as described in Eq. (3c). $$\Delta$$$$\hbox {phase}_{{ref}}$$ grows exponentially with wavelength, reaching $$\infty$$ at $$\lambda _{gr}$$ contributes to $$\Delta$$phase approaching $$\infty$$ in the subsequent grating resonance band, as illustrated in Fig. 3f. The monotonically decreasing $$\Delta$$$$\hbox {phase}_{{inc}}$$ counteracts the exponentially increasing $$\Delta$$$$\hbox {phase}_{{ref}}$$, resulting in a concave-up curve, as shown in the inset of Fig. 3f, with a minimum occurring at $$\lambda$$ = 453 nm. As expected, upon scrutinizing the TE and TM plots in both Fig. 3c,f, it becomes evident that $$\Delta$$$$\hbox {phase}_{{op}}$$ remains independent of polarization for both reflection and transmission.

In the grating resonance band, a resonant peak in $$\Delta$$phase can be observed (the peak is present but not visible in the plot for TE) due to contribution from $$\Delta$$$$\hbox {phase}_{{gr}}$$ of grating resonance at the $$\lambda _{gr}$$ = 576.8 nm. For transmission, $$\Delta$$$$\hbox {phase}_{{gr}}$$ for TE and TM differs, as illustrated in Fig. 3d by the difference $$\Delta$$$$\hbox {phase}_{TM-TE}$$. However, in reflection, $$\Delta$$$$\hbox {phase}_{{gr}}$$ for TE and TM is identical, as can be inferred from Fig. 3g. We attribute this phenomenon to TM exhibiting phase-reversal resonance, while TE exhibits phase resonance. In reflection, both the phase and phase-reversal resonance tend to $$+\infty$$ on the left side of $$\lambda _{gr}$$, while the expected mismatch on the right side doesn’t exist due to the cutoff at $$\lambda _{gr}$$. Therefore, there’s no difference between the TE and TM $$\Delta$$$$\hbox {phase}_{{gr}}$$ for reflection. However, in transmission, the phase-reversal resonance tends to $$-\infty$$, whereas the phase resonance tends to $$+\infty$$, leading to a negative spike in $$\Delta$$$$\hbox {phase}_{TM-TE}$$ on the left side of $$\lambda _{gr}$$. On the right side of $$\lambda _{gr}$$, both tend to $$+\infty$$, with the difference saturating into the adjacent plasmonic resonance band. In the plasmonic resonance band, this difference saturates into a plasmonic phase value $$\Delta$$$$\hbox {phase}_{pr} = 1.2 \times 10^{-3}$$ radians. Therefore, $$\Delta$$phase for any wavelength is the sum of one or more of the three components: $$\Delta$$$$\hbox {phase}_{{op}}$$, $$\Delta$$$$\hbox {phase}_{{gr}}$$, and a constant $$\Delta$$$$\hbox {phase}_{{pr}}$$, as outlined in Eq. () for transmission and reflection, respectively.

The $$\Delta$$$$\hbox {phase}_{{op}}$$ predominates over $$\Delta$$$$\hbox {phase}_{{gr}}$$ or $$\Delta$$$$\hbox {phase}_{{pr}}$$ for all $$\lambda$$ except in the vicinity $$\lambda _{gr}$$. However, within that wavelength range, intensity is very small (|*T*| = 0.56%). Consequently, for further discussion in this work, we utilize the $$\Delta$$$$\hbox {phase}_{{op}}$$ to monitor $$\Delta$$RI. $$\Delta$$$$\hbox {phase}_{{op}}$$ offers the advantage of independence in choosing the operational wavelength $$\lambda _{i}$$. In contrast, $$\Delta$$$$\hbox {phase}_{{gr}}$$ or $$\Delta$$$$\hbox {phase}_{{pr}}$$ necessitate selecting $$\lambda _{i}$$ based on the chosen $$P_G$$ and/or incident angle or employing specific $$P_G$$ and/or incidence angle based on $$\lambda _{i}$$. With the freedom to choose $$\lambda _{i}$$, we propose selecting the wavelength based on the sensitivity of the electrolyte’s RI to changes in concentration and absorbance of the electrolyte, opting for a minimum possible $$\lambda _{i}$$ for transmission and a maximum possible $$\lambda _{i}$$ for reflection mode. For the $$[\hbox {Fe(CN)}_6]^{3-}$$ / $$[\hbox {Fe(CN)}_6]^{4-}$$ solution mixture, absorption is significant in the blue wavelength range, peaking at 420 nm, and extends beyond blue wavelengths with multiple peaks in the UV range, but it exhibits minimal absorption in the green wavelength range^49^. Furthermore, since this is an imaging-based optical readout, and considering that the human eye is more sensitive to green and the Bayer color filter array in digital cameras has twice as many green filters as red or blue filters, we chose a green wavelength of $$\lambda _{i}$$ = 532 nm for this study. This choice ensures minimal interference from absorbance while maximizing the $$\Delta$$phase detection sensitivity. Additionally for this wavelength, both polarizations exhibit similar intensity and somewhat comparable $$\Delta$$phase values. The results and conclusions at $$\lambda _{i}$$ remain valid for all other wavelengths following scale adjustment based on $$\Delta$$phase at the alternate $$\lambda _{i}$$ versus $$\Delta$$phase at $$\lambda _{i}$$ = 532 nm. Additionally, as $$\Delta$$$$\hbox {phase}_{{op}}$$ is independent of polarization, further simulations were polarization insensitive.

**Fig. 4:**
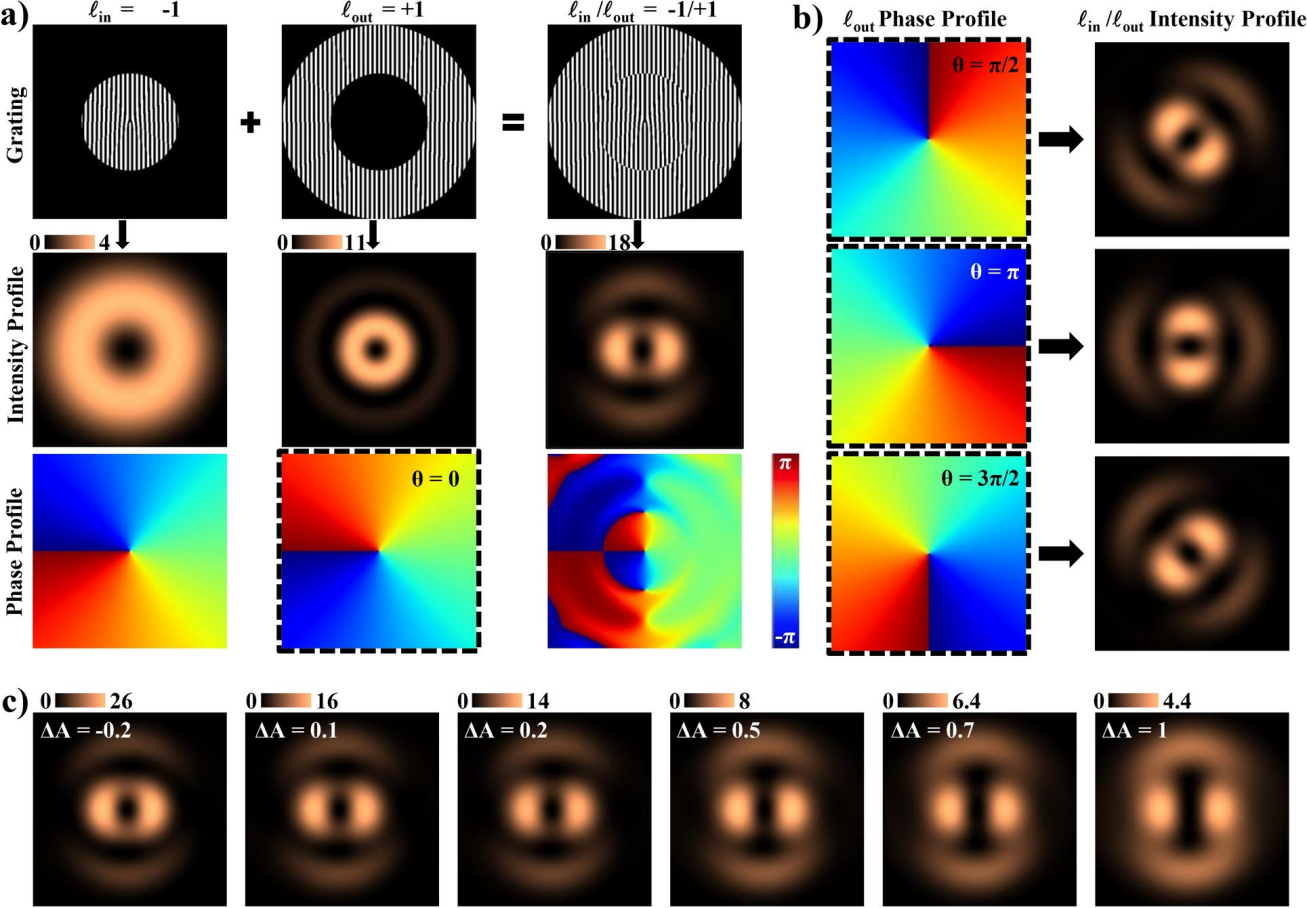
The figure illustrates how a grating can be used for on-chip interference to detect changes in the optical phase induced by the EC reaction. The grating is divided into two concentric regions, each responsible for generating one of the two interfering vortex beams with different charges. Any alteration in the phase of one beam will result in a change in the intensity profile of the interference. (**a**) Shows the intensity and azimuthal phase profiles of the $1^{st}$-DO individually for each segment of the grating. The inner segment features a charge − 1 fork-grating (first column), while the outer segment features a charge + 1 fork-grating (second column). Subsequently, the third column presents a resultant COV beam for both segments combined. (**b**) shows the effect of change in phase, denoted as $\theta$, of the $1^{st}$-DO from the outer grating segment on the intensity profile of the resultant beam. The resultant intensity profile can be observed to undergo a rotation of $\theta$/2. (**c**) shows the effect of the difference in absorbance, denoted as $\Delta$A, between the outer and inner grating segments for a constant phase difference of $\theta = 0$. The resultant change in the intensity profile is insignificant for small $\Delta$A values. The rotational orientation of the intensity profile can be observed to be preserved for all $\Delta$A values.

Although not elaborated upon in this study, both $$\Delta$$$$\hbox {phase}_{{gr}}$$ and $$\Delta$$$$\hbox {phase}_{{pr}}$$ exhibit polarization dependence for the case of transmission and can be utilized in scenarios where the EC information resides in the polarization of the beam. This could particularly be relevant when the reactant and product of a reaction possess differences in chirality. Interestingly, in the plasmonic band, $$\Delta$$$$\hbox {phase}_{TM-TE}$$ is wavelength-independent, and the amplitude demonstrates a reverse trend for TE and TM. For TE, the amplitude steadily decreases to zero, whereas for TM, the amplitude gradually increases from approximately zero to a peak at $$\lambda _{pr}$$ = 612 nm.

### Huygen’s simulation

The $\Delta$phase in a beam can be measured by interfering it with a reference beam not having $\Delta$phase. A beam passing through the RE can serve as the reference beam. In an EC setup, reactions occur at the WE, modifying the concentration profile of electrochemically active species and resulting in a $\Delta$RI in the vicinity of the WE. In contrast, the RE ideally maintains a stable concentration profile, ensuring a constant reference potential, and thus, no $\Delta$RI in its vicinity.

To deduce $\Delta$phase through interference, we have chosen to employ an on-chip interference method instead of the traditional interferometric setup. Figure 4a shows the design of a hybrid binary fork grating (hBFG) studied in detail in our previous report^50^. This design involves dividing a single grating into two concentric segments, each with a fork grating of different charge: $\ell _{in} = -1$ for the inner segment $\hbox {hBFG}_{{in}}$ and $\ell _{out} = +1$ for the outer segment $\hbox {hBFG}_{{out}}$. Upon combination, these segments form the hBFG, generating coaxially superposed interfering vortex beams in the DOs of the grating. These beams constitute a composite optical vortex (COV) beam characterized by a central null surrounded by peripheral nulls, whose orientation is determined by the phase difference between the two interfering beams. Consequently, variations in the incident beam phase between the two segments lead to the rotation of the intensity profile of the COV beam, as illustrated in Fig. 4b. The difference in absorbance ($\Delta$A) between the two segments, i.e., the absorbance of $\hbox {hBFG}_{{out}}$ minus the absorbance of $\hbox {hBFG}_{{in}}$, leads to a change in the intensity profile of the COV beam, as illustrated in Fig. 4c. However, this change is significant only for high $\Delta$A values, such as 1, i.e., the magnitude of *T*/$\Gamma$ of one region is 10 times that of the other. Moreover, it can be observed that $\Delta$A has no effect on the rotation of the intensity profile of the COV beam.

In this study, we propose electrically isolating the two segments of an hBFG, using one segment as a WE and the other as a RE. This setup enables the capture of $\Delta$phase resulting from reactions at the WE, manifested in the rotation ($\theta$) of the COV beams. The relationship between the $\Delta$phase and the angle of rotation $\theta$ of the COV beam is described by Eq. (4). On substituting the value for $\ell _{in}$ and $\ell _{out}$ of the hBFG shown in Fig. 4a, in this equation gives $\theta = \Delta$phase/2, as confirmed by the observed rotation of the intensity profile in Fig. 4b.

4 $$\begin{aligned} \theta = \Delta \text {phase}/ (\ell _{out}-\ell _{in}) \end{aligned}$$

**Fig. 5:**
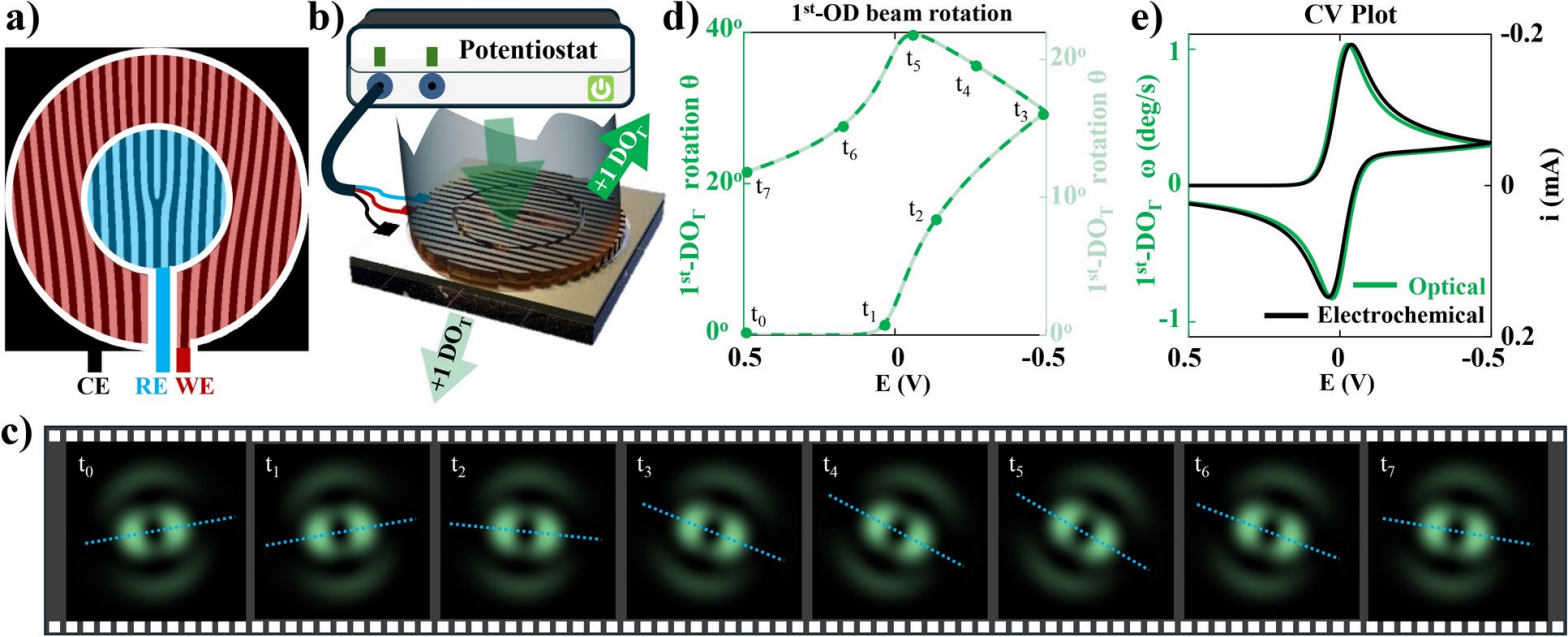
The figure illustrates how an EC experiment and an interference experiment can be simultaneously performed on the grating, allowing the EC reaction to be visually monitored as the rotation of COV beams. (**a**) Shows the hBFG electrically isolated into three regions to be used as WE, RE and CE electrodes. (**b**) Shows the simulation setup schematic with different regions of the hBFG connected to the appropriate pins of a potentiostat. The setup collects the $1^{st}$-DO from a normally incident beam of green wavelength 532 nm. (**c**) Shows the orientation of the COV beam intensity profile at different time instants from $t_0$ to $t_7$, as marked in (**d**). (**d**) shows the plot of **E** vs $1^{st}$-DO intensity profile rotation angle $\theta$ for both reflected and transmitted COV beam. (**e**) Shows the optically obtained CV plot ($\hbox {CV}_O$) as of rate of rotation $\omega = d\theta /dt$ vs **E** on the left y-axis whereas conventional CV plot ($\hbox {CV}_E$) as **i** vs **E** on the right y-axis.

**Fig. 6:**
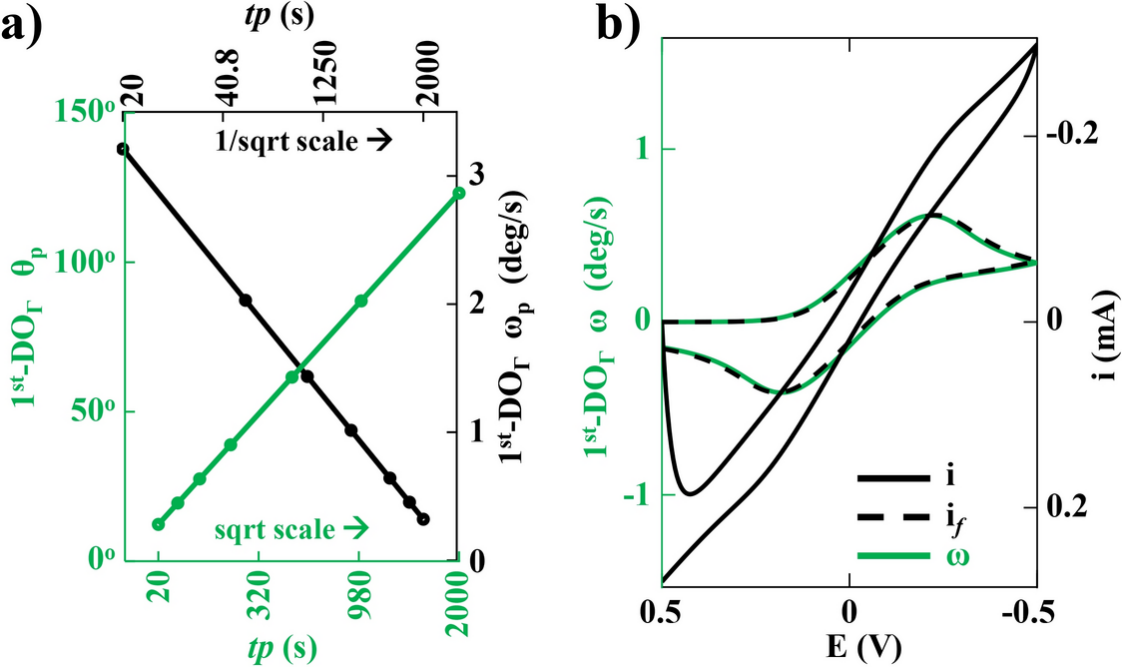
(**a**) Variation of peak rotation $\theta _p$ and peak rotation rate $\omega _p$ with CV time period *tp*, (**b**) a comparison between $\hbox {CV}_E$ and $\hbox {CV}_O$ plot for a non-faradaic component dominated EC System.

### O-EC simulation

Figure 5a illustrates the schematic of the hBFG as the proposed structured electrode with three regions: the inner region highlighted in blue, the outer region highlighted in red, and the regions adjacent to the grating, but not part of it, highlighted in black. The black region serves as the CE, while the red and blue regions can be used as WE or RE. The choice of WE and RE does not significantly impact the final result since it depends on the relative phase difference between the two regions. In this work, we have chosen the red outer region adjacent to the CE as the WE and the inner blue region as the RE. The on-chip interference on the hBFG electrode simplifies the O-EC setup by imposing minimal requirements on optical components and alignment, while also minimizing alterations to the EC setup or disturbance to the EC measurement itself. The O-EC setup is shows in Fig. 5b which comprises a potentiostat for driving the EC experiment, hBFG as structured electrodes for generating COV beams, i.e., a structured electrode for generating structured beams, and an optical source and the collection of the optical response in the $1^{st}$-DO. The optical response manifests as a video showing the rotation of the COV beam during the progression of an EC experiment. A video demonstrating one cycle of a CV experiment alongside the corresponding rotation of the COV beam can be found as Supplementary Video S2 . Snapshot images from the video at eight different time instants over one cycle of a CV experiment are shown in Fig. 5c. These eight time instants have been marked in the plot in Fig. 5d which shows $\theta$ versus applied potential **E**. It is noteworthy that the reflection and transmission plots contain exactly the same information and differ only in scale. Additionally, the optical response is proportional to the total reaction, whereas the conventional electrical response is proportional to the rate of reaction. Thus for a direct comparison, Fig. 5e presents the plot of rate of rotation $\omega = d\theta /dt$ on the left y-axis and the electrical CV on the right y-axis, demonstrating a one-to-one match between the electrical and optical voltammograms.

There is a key difference between the electrical and optical responses. The electrical response at any time **t** is proportional to the total reaction over the WE area $\textbf{A}$ per unit time. In contrast, the optical response is proportional to the total reaction over a length of time **t** per unit area. Therefore, extending the duration of the EC experiment can amplify the optical response by allowing more product accumulation near the WE. This is illustrated in Fig. 6a, which shows the difference in the plots of peak rotation $\theta _{p}$ and peak rate of rotation $\omega _{p}$ versus the time period *tp* of the EC experiment. As previously demonstrated for CV, the plot of $\omega$ resembles **i**. The peak current ${\textbf {i}}_p$ is proportional to the square root of the scan rate $\nu$ defined in Supplementary S1 as Table ST1^1,26^, thus proportional to $1/\sqrt{tp}$. Consequently, $\omega _{p}$ was found to be proportional to $1/\sqrt{tp}$, shown as the black line in Fig. 6a. On the other hand, $\theta$ represents the total reaction over a time period, thus $\theta _{p}$ was observed to be proportional to $tp \times 1/\sqrt{tp} = \sqrt{tp}$ , shown as the green line in the same plot in Fig. 6a but on a different axis. Aside from the second key difference between electrical and optical responses - where both $\theta$ and $\omega$ were found to be independent of the WE area - both responses showed a linear trend with concentration and the square root of the diffusion coefficient, similar to what is observed in conventional electrical CV experiments.

Figure 6b shows the comparison of the voltammogram in the presence of large non-faradaic current and solution resistance. The CV plot, depicted by the solid black line, appears highly distorted, making it extremely challenging to extract the desired faradaic current, as shown by the black dotted curve within the same plot. However, since the optical readout corresponds to the concentration of the reactant and the product, $\omega$ is solely proportional to the faradaic current. Therefore, the optical readout facilitates the direct recovery of the faradaic current.

## Discussion

Optical voltammogram offers several advantages in EC analysis. Firstly, it enables the direct recovery of faradaic current, providing valuable insight into EC processes. While the issue of ohmic drop is not directly resolved, the structured electrode is highly scalable and miniaturizing it to a microelectrode can mitigate this problem^33^. Furthermore, this method has the capability to mitigate the influence of environmental fluctuations, such as temperature variations occurring across the region encompassing both the working electrode (WE) and the reference electrode (RE). Also, this method is well-suited for scenarios involving slow reactions or small electrodes with minimal current, where traditional EC CV may be limited. Here, the optical response offers an integrated measurement over time, enhancing sensitivity, particularly when integrated for longer durations. Furthermore, when compared to SPR imaging, our approach eliminates the need for complex optical setup and alignment, allowing researchers to focus on chemical effects. It is adaptable to various grating sizes and incident wavelengths, providing flexibility in experimental design by allowing researchers to choose wavelengths where RI changes are maximal for a given analyte and minimal in terms of absorbance. As $\hbox {CV}_O$ directly correlates with changes in bulk properties, it is particularly useful for tracking reaction pathways, especially in secondary homogeneous reactions or for monitoring intermediates in multi-step reactions.

Despite these advantages, there are some limitations to consider. Changes in RI due to concentration variations of unwanted species may introduce measurement inaccuracies. Furthermore, in the case of fast reactions, the small reaction products and slow diffusion can adversely impact optical voltammetry, reducing its effectiveness. When compared to SPR imaging, our method may lack sensitivity to interface changes, and scanning may be required to generate an image.Another notable limitation is that the resolution and limit of detection depend on the ability to resolve the rotation, which degrades with the quality of the beam profile.

Looking ahead, the proposed method holds promise for a range of future applications and developments. Firstly, it can be adapted to track EC reactions through alternative optical properties such as absorbance or polarization, broadening its utility beyond conventional EC analysis. Additionally, the integration of structured electrodes with optical readouts opens up possibilities for creating arrays of shorted WEs, enabling localized EC reaction imaging for spatially resolved EC information. Moreover, our approach can be extended to study biochemical reactions where EC reaction is not involved, offering a versatile tool for diverse research fields. Finally, it can be utilized to investigate light-induced reactions, encompassing both optically driven reactions and those probed electrochemically, thereby providing a comprehensive platform for exploring a wide range of chemical processes. Ultimately, these future perspectives highlight the adaptability and potential of our proposed method to address various research needs and advance our understanding of chemical phenomena.

## Methods

The CV simulations involved finite difference time domain (FDTD) modeling of the Eq. (S.5) in Supplementary S1 and has been discussed in detail in our previous report^26^. The phase and intensity of the $1^{st}$-DO of the ut-MGM were simulated using the RCWA method^51^, individually for $\hbox {hBFG}_{{in}}$ and $\hbox {hBFG}_{{out}}$, with parameters specified in Supplementary S1 as Table ST1. Whereas, the transverse phase and intensity profile of the $1^{st}$-DO of hBFG, with parameters specified in Supplementary S1 as Table ST1, were calculated based on the Huygens principle, as discussed in detail in our previous report^50^.

All the simulations were conducted in an online Python notebook^52^. All data and functionality related to CV, RCWA and Huygens simulations were encapsulated in a class named *CyclicVoltammetry*, *RCWA* and *diffraction*, respectively. EC input parameters such as **E**(t) and initial concentration $c_{ox}^*$ must be specified at the *CyclicVoltammetry* class object instantiation. Physical parameters of the on-chip O-EC cell such as unit cell dimension and material of the hBFG, incidence wavelength and incidence angle and simulation parameters such as number of RCWA layers and thickness of each layer has to be specified at the *RCWA* class object instantiation. The inner $r_{in}$ and outer $r_{out}$ radius along with the $\ell _{in}$ and $\ell _{out}$, screen dimension $S_{sz} \times S_{sz}$ and distance R from hBFG has to be specified during the *diffraction* class object instantiation. Post instantiation O-EC simulation was performed as described in Supplementary S1 as Algorithm SA1.

## Conclusion

Our study introduces a novel approach for O-EC analysis that overcomes several limitations associated with conventional EC methods. By integrating structured electrodes with optical readouts, we achieve direct measurement of the faradaic current without the need for extensive post-processing, offering a streamlined and efficient analytical technique. Moreover, our on-chip interference method simplifies the experimental setup, eliminating the requirement for intricate optical alignment and facilitating ease of use. Looking ahead, our proposed method holds significant potential for further development and application. Firstly, it can be coupled with various voltammetric techniques and operated at multiple wavelengths, enabling versatile analysis of EC processes. Additionally, the design lends itself to the formation of microelectrode arrays with a single electrically-shorted contact for all WEs, all REs and all CEs, paving the way for local EC reaction monitoring. Overall, our findings demonstrate the promise of O-EC analysis for advancing our understanding of chemical reactions and facilitating future research endeavors in the field. By integrating optical techniques with conventional EC analysis, we broaden the scope of investigation, providing complementary insights and data.

## Supplementary Information

Supplementary Video 1.

Supplementary Information.

## Data Availability

All data generated or analyzed during this study are included in this published article and its supplementary information files.
