# Supplementary material for: A simulation-based analysis of optical read-out for electrochemical reactions using composite vortex beams
Source: Sci Rep. 2024 Sep 27;14:22218. doi: 10.1038/s41598-024-72701-8 (PMC11437161; doi:10.1038/s41598-024-72701-8)
Supplement: Supplementary file 2 — Supplementary Information. [file 41598_2024_72701_MOESM2_ESM.pdf]

Supplementary Information for: A Simulation-Based Analysis of Optical Read-Out for Electrochemical Reactions Using Composite Vortex Beams

Nirjhar Kumar[1,*], Ankit Arora[1], and Ananth Krishnan[1]

[1]Centre for NEMS & Nanophotonics (CNNP) and Department of Electrical Engineering, Indian Institute of Technology Madras, Chennai-600036, India
[*]Corresponding author: kr.nirjhar@gmail.com

RI of Electrolyte

The refractive index (RI) can be determined from the concentration of each species in the electrolyte using the Lorentz-Lorenz equation given in (S.1) where ρ_k and α_k represent the number density and polarizability of the kth species in the electrolyte[1].

$$\frac{RI^2-1}{RI^2+2} = \frac{1}{3\varepsilon_0}\sum_k \rho_k \alpha_k \tag{S.1}$$

For an aqueous solution sup containing species ox and red in water w, (S.1) can be expanded as follows:

$$\frac{RI_{sup}^2-1}{RI_{sup}^2+2} = \frac{1}{3\varepsilon_0}[\rho_w\alpha_w + \rho_{ox}\alpha_{ox} + \rho_{red}\alpha_{red}] \tag{S.2}$$

For low concentrations, ρ_w in (S.2) can be assumed to be same as that of the pure water given in (S.2a), whereas ρ_{ox} and ρ_{red} are as given by Eqs. (S.2b-c) where ε_0 is the free space permittivity , N_A is the Avogadro constant and factor of 1000 is required to convert the concentration from moles per liter to moles per cubic meter.

$$\frac{RI_w^2-1}{RI_w^2+2} = \frac{1}{3\varepsilon_0}\rho_w\alpha_w \tag{S.2a}$$

$$\rho_{ox} = 1000N_A c_{ox} = 1000N_A c_{ox}^*\gamma \tag{S.2b}$$

$$\rho_{red} = 1000N_A c_{red} = 1000N_A c_{ox}^*(1-\gamma) \tag{S.2c}$$

Substituting the above equation Eqs. (S.2a-c) into (S.2):

$$\frac{RI_{sup}^2-1}{RI_{sup}^2+2} = \frac{RI_w^2-1}{RI_w^2+2} + \frac{1000N_A C_{ox}^*}{3\varepsilon_0}[\gamma\alpha_{ox} + (1-\gamma)\alpha_{red}] \tag{S.3a}$$

Additionally, for low concentrations where the values of RI_{sup} and RI_w are close to each other:

$$\frac{RI_{sup}+1}{RI_{sup}^2+2} \approx \frac{RI_w+1}{RI_w^2+2} \tag{S.3b}$$

By dividing both sides of (S.3a) by the corresponding sides of (S.3b):

$$RI_{sup} - 1 = RI_w - 1 + \frac{RI_w^2+2}{RI_w+1}\frac{1000N_A C_{ox}^*}{3\varepsilon_0}[(1-\gamma)(\alpha_{red}-\alpha_{ox}) + \alpha_{ox}] \tag{S.3}$$

Thus, the RI profile can be expressed in terms of γ for a given C_{ox}^*, as shown in (S.4).

$$RI_{sup} = RI_w + 36.572\times10^{36}C_{ox}^*[(1-\gamma)(\alpha_{red}-\alpha_{ox}) + \alpha_{ox}] \tag{S.4}$$

Simulation Parameter Table

Simulation parameters used in this study is as given below:

Table ST1. Simulation parameters

Parameters	Symbols	Values	Unit
**CV Simulations:**			
Temperature	T	298	K
WE radius	r_{out}	0.3	cm
Initial Concentration	c^*_{ox}	10	mM
Diffusion coefficient	D	6.5×10^{-6}	cm^2/s
time period	tp	200	s
CV initial potential	$\mathbf{E}_i$	0.5	V
CV switching potential	$\mathbf{E}_f$	-0.5	V
Scan rate = $\frac{2\lvert\mathbf{E}_f-\mathbf{E}_i\rvert}{tp}$	ν	20	mV/s
Bulk $\approx 6\sqrt{D\times tp}$	x_{bulk}	2.16	mm
DL Capacitance	C_{dl}	20	mF/cm^2
Series resistance	R_s	1	kΩ
Parallel DL resistance	R_p	1	kΩ
RI water	RI_w	1.3337	RIU
$K_3Fe(CN)_6$ polarizability	α_{ox}	15.274×10^{-40}	$C\ m^2\ V^{-1}$
$K_4Fe(CN)_6$ polarizability	α_{red}	22.633×10^{-40}	$C\ m^2\ V^{-1}$
Discretization in space x	N_x	50	samples
Discretization in time t	N_t	401	samples
**RCWA Simulations:**			
No of layers = N_x+1	N_L	51	samples
Dielectric Layer thickness = $\frac{x_{bulk}}{N_x-1}$	t_{el}	44.08	μm
Metal Layer Thickness	t_{ml}	30	nm
Superstrate RI	RI_{sup}	1.33	RIU
Substrate i.e glass RI	RI_{sub}	1.52	RIU
Metal RI	RI_{Au}	Au_{palik}	RIU
Grating Period	P_G	500	nm
Grating duty cycle	dc_G	50	%
**Huygen's Simulations:**			
grating inner radius	r_{in}	7.5	μm
grating outer radius	r_{out}	15	μm
screen distance	R	3	mm
screen size	S_{sz}	250	μm
incidence wavelength	λ_i	500	nm
incidence beam waist	w_0	18	μm

CV difference equation used in simulation

The CV simulations for a diffusion-limited, 1e-RER were implemented using finite difference time domain (FDTD) modeling of a diffusion equation for the reactant concentration ($c_{ox}(x,t)$) described by (S.5).

$$\frac{\partial c_{ox}}{\partial t} = D\left(\frac{\partial^2 c_{ox}}{\partial x^2}\right) \tag{S.5}$$

The boundary conditions of (S.5), governed by Nernst equilibrium at $x = 0$ and constant at $x_{bulk} = 2.16$ mm are as given below:

$$c_{ox}(x=0,t) = \frac{c^*_{ox}}{1+\exp\left(-\frac{\mathbf{E}(t)}{RT/F}\right)} \tag{S.5a}$$

$$c_{ox}(x=2.16\text{ mm},t) = c^*_{ox} \tag{S.5b}$$

Additionally, a uniform initial concentration profile assumed for the initial condition is given by:

$$c_{ox}(x,t=0) = c^*_{ox} \tag{S.5c}$$

Algorithm

Algorithm SA1 Algorithm for O-EC simulation

for $t_n = 0 \rightarrow t_N$ **do**

- **step 1: Execute *runVoltametryNextVSample(...)*** which calculate the $c_{ox}[x]$ and $RI_{sup}[x]$ at t_n and subsequently returns $\mathbf{i}[t_n]$ and $\mathbf{i}_f[t_n]$
- **step 2: Update the dielectric RI** of each RCWA layer based on $RI_{sup}[x]$
- **step 3: Execute *rcwa_multi_tm(...)*** which returns 1st-DO $\Gamma[t_n]$ and $T[t_n]$
- **step 4: Update the incident phase** over hBFG_{out} based of angle($\Gamma[t_n]$) or angle($T[t_n]$) while maintaining the incident phase over hBFG_{in} same as at t_0
- **step 5: Execute *run_huygens_construct()*** which returns the 1st-DO intensity profile Frame[t_n]

end forstep 6: Plot and analyse $\mathbf{i}[t]$ vs $\mathbf{E}[t]$ alongside Frame[t] compiled into a video.

Supplementary Video S2 caption

The plot in the left half of the video illustrates a conventional cyclic voltammetry (CV) plot for one CV cycle (*tp* = 200 s), depicted by the red curve. Simultaneously, the blue curve represents the corresponding rotation of the composite optical vortex (COV) beam intensity profile. This half of the video has been sped-up and synchronized with the sped-up video of the COV beam intensity profile displayed in the right half of the video, enhancing visualization of COV beam rotation with CV.

References

1. An, N., Zhuang, B., Li, M., Lu, Y. & Wang, Z.-G. Combined theoretical and experimental study of refractive indices of water–acetonitrile–salt systems. *The J. Phys. Chem. B* **119**, 10701–10709 (2015).
